# Identification of a novel pathogenic *TBCK* variant in a Chinese patient with infantile hypotonia with psychomotor retardation and characteristic facies type 3 (IHPRF3): a case report

**DOI:** 10.1186/s12887-022-03672-w

**Published:** 2022-10-22

**Authors:** Hao-Yi Tan, Bin Wang, Yuan-Zong Song

**Affiliations:** 1 Department of Pediatrics, The First Affiliated Hospital, Jinan University, Guangdong, 510630 China; 2 Department of Pediatrics, Zhujiang Hospital, Southern Medical University, Guangdong, 510280 China

**Keywords:** IHPRF3, *TBCK*, Genetic mutation, Child, Hypotonia

## Abstract

**Background:**

Infantile hypotonia with psychomotor retardation and characteristic facies type 3(IHPRF3) (OMIM #616,900) is an autosomal recessive disorder caused by biallelic pathogenic variants of the *TBCK* gene, and to date, this disease was reported rather limitedly in number and all described cases were Caucasians.

**Case presentation:**

This paper reported the clinical and genetic features of a Chinese patient with IHPRF3. The patient was a 15-month-old male with global developmental delay, profound hypotonia, and typical facial dysmorphic features including mildly coarse facial appearance, hypertelorism, tented upper lip, exaggerated Cupid’s bow, macroglossia and arched eyebrows. Magnetic Resonance Imaging (MRI) analysis of the brain revealed slightly widened bilateral ventricles and subarachnoid space. On genetic analysis, the patient was homozygous for a novel *TBCK* variant c.247C > T(p.Arg83Ter). The parents were both carriers without any positive symptoms or signs. With an extremely low frequency (0.0000082) in Exome Aggregation Consortium, the variant has not been reported in any other databases or official literatures, and was diagnosed to be pathogenic according to the American College of Medical Genetics and Genomics(ACMG) standards and guidelines. Neurorehabilitation training did not work well and the long-term prognosis remained to be observed.

**Conclusions:**

This study reported the clinical and molecular features of the first non-Caucasian patient with IHPRF3 arising from a novel homozygous *TBCK* mutation, which provided a novel molecular marker for the definite diagnosis of IHPRF3 patients and for its genetic counseling and prenatal diagnosis in the affected families.

## Background

Infantile hypotonia with psychomotor retardation and characteristic facies type 3 (IHPRF3) is an infantile encephalopathy syndrome caused by biallelic pathogenic mutations in the *TBCK* (TBC1 domain containing kinase) gene, which is located on chromosome 4 with a total of 26 exons and encodes an 893-aa protein with kinase, TBC (Tre-2, Bub2, and Cdc16) and Rhodanese homology domains [1]. *TBCK* is involved in the regulation of cell proliferation, cell growth, and actin organization by making an impact on the expression of mammalian target of rapamycin (mTOR) complex (mTORC) components at the transcriptional level. The main clinical manifestations included systemic growth retardation, special facial features, drug-resistant epilepsy, and chronic respiratory failure. Other frequently occurring phenotypes included poor language expression ability, hypotonia, visual impairment, hearing impairment and bone metabolism disorders [2].

The first case of IHPRF3 was reported in 2008 [3], and to date, this disease was still a rare condition with patients extremely limited in number. So far as we know, only 45 such patients have been reported worldwide and all of them were Caucasians [4–6]. Hence, the clinical and molecular characteristics of this disease remain open for investigation, especially in non-Caucasian populations. Herein, we reported the clinical and genetic presentations of the first IHPRF3 patient in Asian population.

## Case presentation

**Fig. 1:**
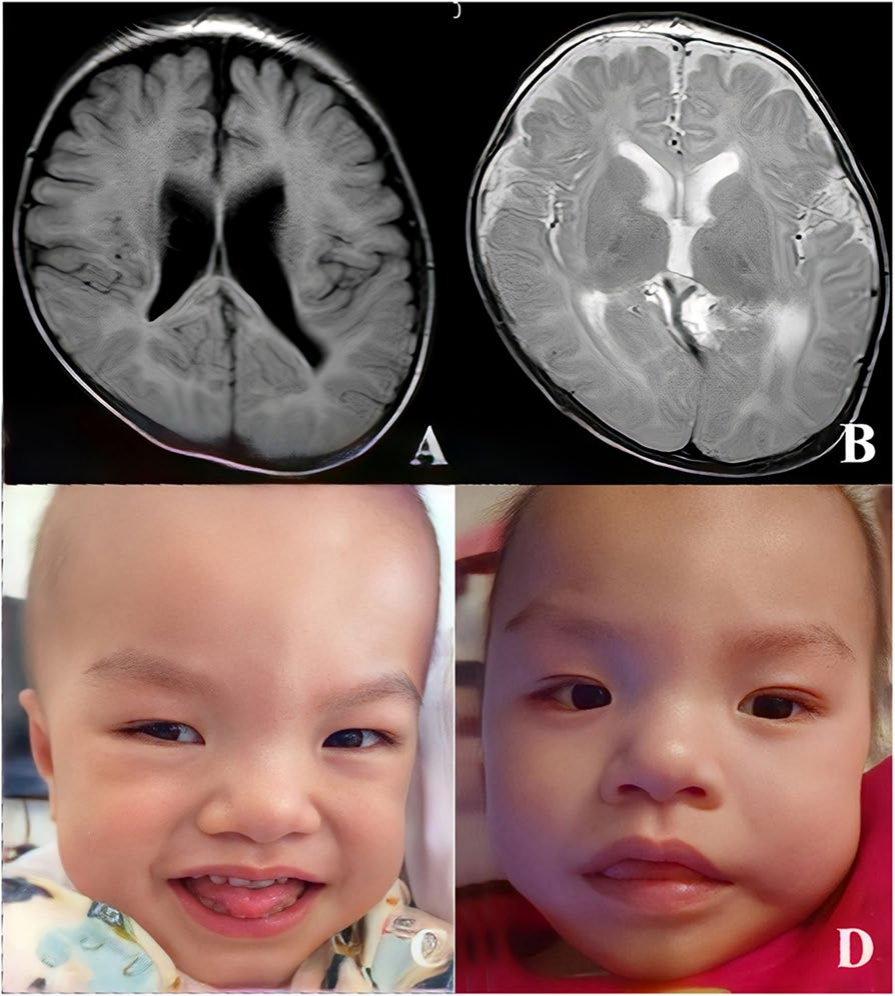
Head MRI Findings and photographs of the dysmorphic features Axial T1-weighted FLAIR (Fluid Attenuated Inversion Recovery) images (**A**) demonstrated widened anterior cerebral longitudinal fissure and enlarged bilateral ventricles. The subarachnoid space in the anterior part of the cerebral hemisphere was also widened. Axial T2-weighted TSE (Turbo Spin Echo) images(**B**) demonstrated the same imaging presentations. The patient had special facial feature including tented upper lip, exaggerated Cupid’s bow, arched eyebrows, macroglossia, hypertelorism and open mouth (**C** & **D**)

The patient was a 15-month-old boy with global developmental delay noticed for 10 months. He was found to be unable to raise his head at 5 months of age when electroencephalogram was normal but brain Magnetic Resonance Imaging (MRI) (Fig. 1) revealed slightly widened bilateral ventricles and subarachnoid space. No abnormalities were found in chromosome karyotype analysis and screening for genetic metabolic diseases. Physiotherapy and rehabilitation training was performed but the effect was not promising. When aged 15 months, he could still neither lift his head nor be seated unsupportedly, and could not call “mom” and “dad” intendedly.

He was born at the gestational age of 38 weeks and one day as the product of a non-consanguineous couple, with a birth weight of 3.75 kg and body length 50 cm that were appropriate for gestational age but with an occipitofrontal head circumference of 38 cm with a Z score + 2.91SD. When aged 6 days, he was admitted into the Division of Neonatology in the Department of Pediatrics of our hospital for 12 days because of neonatal hyperbilirubinemia and pneumonia, where low muscular tension and muscle strength were noticed. Because of the lack of other positive symptoms or signs, the family decided to observe his development without any treatment.

Physical examination at referral revealed a body weight of 10 kg, body length 80 cm and head circumference 48.5 cm(+ 1SD). No jaundice was observed in the skin and sclera. Special facial features were observed, including mildly coarse facial, hypertelorism, tented upper lip, exaggerated Cupid’s bow, macroglossia, open mouth and arched eyebrows (Fig. 1). No stridor, crackles or crepitus could be heard in the two lungs, and the heart sound was normal without any murmurs. There was no abdominal distention, and the liver and spleen were non-palpable. Marked hypotonia was found especially at the lower limbs. Abdominal wall reflex was weak, and neither achilles nor knee tendon reflex could be drawn. The ribbed arch valgus and pectus excavatum were observed. The palm and sole prints were deep, and horizontal wrinkles could be seen on both knees. The testis had not descended into the scrotum.

The Gesell Developmental Schedules (GDS) analysis was conducted, and a score of 8.9 for gross motor function was got, along with the scores 23.7 for fine motor function, 26.6 for adaptive behavior, 26.6 for language ability and 26.6 for social skill, indicating global developmental delay.

**Fig. 2:**
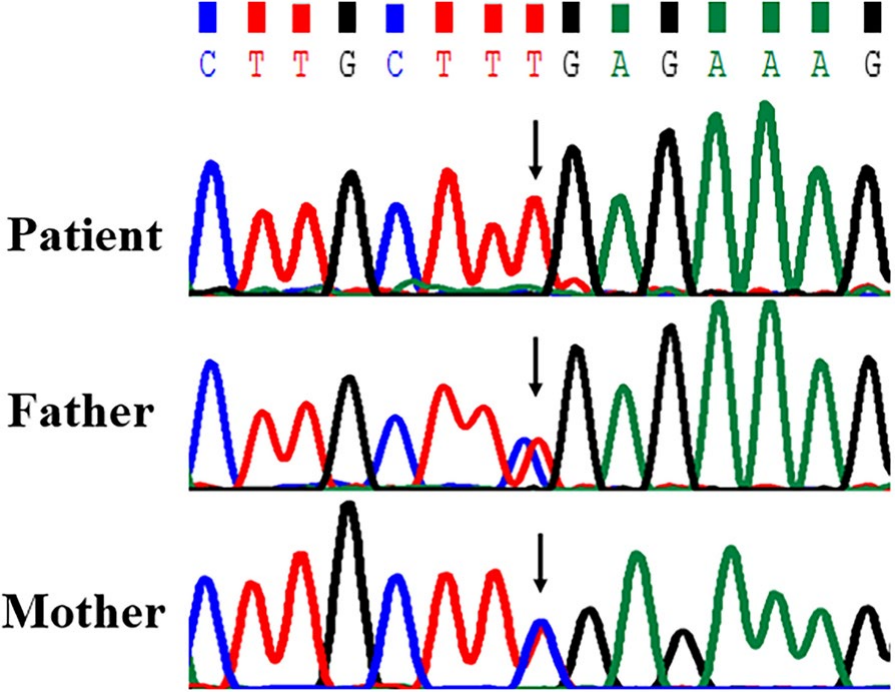
Sanger sequencing results for the patient and his parents The child was homozygous for the *TBCK* mutation c. 247C > T, and both parents were carriers

Considering his clinical presentations, genetic neuromuscular disease was highly suspected and hence Next Generation Sequencing (NGS) was carried out to explore the underlying genetic etiology. As a result, NGS detected a homozygous *TBCK* mutation c.247C > T(p.Arg83Ter) in the patient, which was a nonsense mutation located in exon 3. Sanger sequencing confirmed that the child was homozygous for this variant, and his healthy father and mother were both carriers (Fig. 2). To the best of our knowledge, the variant,with an extremely low frequency (0.0000082) in Exome Aggregation Consortium, has not been reported in any other databases or official literatures.

Based on the clinical and genetic findings above, the patient in this study was diagnosed with IHPRF3.Neurorehabilitation training was continued, but on a follow-up at his age of 17 months, no significant improvement was observed in the clinical manifestations such as growth retardation. The long-term prognosis remained to be observed.

**Fig. 3:**
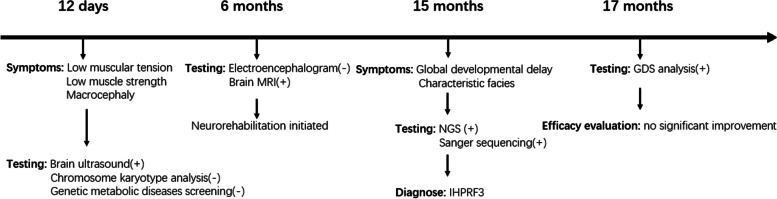
Patient information in a timeline

Figure 3 illustrated the relevant information of the patient in a timeline.

## Discussion and conclusions

In this study, the patient had phenotypes of severe global developmental delay, while low muscle tension and strength, special facial features and macrocephaly at birth were noticed. Although positive, the brain MRI findings was not severe or specific. On genetic analysis, the child was a homozygote of the *TBCK* mutation c.247C > T which caused transcription to stop at 83-aa, giving rise to a truncated protein p.Arg83Ter,which met the PVS1 criteria in the American College of Medical Genetics and Genomics (ACMG) standard and guidelines [7]. Both parents were carriers of this mutation without positive signs or symptoms, which reached the PP1 criteria. Moreover, the variant was found with an extremely low frequency (0.0000082) in Exome Aggregation Consortium(EAC) and no matching records found in Exome Variant Server (EVS) or official literatures, which was a PM2 evidence. Hence, the variant c.247C > T(p.Arg83Ter) was diagnosed as “pathogenic” and the patient was thus diagnosed to suffer from IHPRF3.

*TBCK* was documented to play an important role in the control of cell proliferation, cell size, and actin-cytoskeleton dynamics by modulation of the mTOR signaling network [1, 8]. And in this case, the patient’s severe global developmental delay might be attributed to dysregulated mTOR signaling which caused his thinner bilateral cerebral cortex shown on brain MRI. The patient also had severe hypotonia, which was not surprising since mTOR mediated key endogenous neuroprotective mechanisms in motoneurons [9]. The patient in this study had an obvious macrocephaly at birth, but when aged 15 months, his head circumference became normal. To date, only four IHPRF3 patients were mentioned with macrocephaly [10]. Whether and how macrocephaly was associated with IHPRF3 remained an issue needing to be addressed.

At present, the treatment of IHPRF3 was mainly symptomatic and supportive, including regular follow-up, rehabilitation training and giving respiratory support if necessary to delay the progression of the disease. Of note, *TBCK* influences the expression of mTOR components at the transcriptional level [11], and therapeutic mTORC1 activation may be a potential strategy to prevent the progression of the disease. Actually, the mTOR pathway could be stimulated by exogenous L-leucine supplementation [12], and leucine supplementation was already clinically available for certain metabolic disorders [10, 12]. However, the prognosis generally appeared to be unfavorable [6]. Delayed motor function and severe hypotonia were common in IHPRF3, which caused a decline in the quality of life and respiratory failure posed a threat to the lives in patients with involvement of respiratory muscles.

In conclusion, this study reported the clinical and molecular features of the first non-Caucasian patient with IHPRF3 arising from a novel homozygous *TBCK* mutation, which provided a novel molecular marker for the definite diagnosis of IHPRF3 patients, and for its genetic counselling and prenatal diagnosis in the affected families.

## Data Availability

The datasets generated and analyzed during the current study are available in the GenBank of International Nucleotide Sequence Collaboration (INSDC) repository (GenBank: OP113122.1).

## Abbreviations

IHPRF3: Infantile hypotonia with psychomotor retardation and characteristic facies type 3
MRI: Magnetic Resonance Imaging
ACMG: American College of Medical Genetics and Genomics
*TBCK*: TBC1 domain containing kinase
mTOR: Mammalian target of rapamycin
GDS: The Gesell Developmental Schedules
NGS: Next Generation Sequencing
EAC: Exome Aggregation Consortium
EVS: Exome Variant Server
FLAIR: Fluid Attenuated Inversion Recovery
TSE: Turbo Spin Echo
INSDC: International Nucleotide Sequence Collaboration
